# Factors associated with readiness to start antiretroviral therapy (ART) among young people (15–24 years) at four HIV clinics in Mulago Hospital, Uganda

**DOI:** 10.4314/ahs.v21i4.14

**Published:** 2021-12

**Authors:** Jonathan Nkalubo, Moureen Mugaba, Ignatius Asasira, Racheal Nakiganda, Florence Namutebi, Nick Ntore Arnaud, Nicholas Kagumba Musisi, Trinity Abasira, Pius Jemba, Racheal Ndyabawe, Rosette Tumuhairwe, Charles Batte, Sabrina Bakeera-Kitaka

**Affiliations:** 1 School of Medicine; 2 School of Biomedical Sciences; 3 School of Health Sciences; 4 School of Public Health; 5 Makerere Lung Institute; 6 Department of Paediatrics and Child Health. Makerere University College of Health Sciences, P.O.Box 7072 Kampala, Uganda; 7 Department of Biochemistry & Sports Sciences, Makerere University College of Natural Sciences, P.O.BOX 7062 Kampala, Uganda

**Keywords:** Antiretroviral therapy Readiness, Young people, Sub-Saharan Africa

## Abstract

**Introduction:**

Globally, the HIV burden continues to rise among young people despite the discovery of ART. This study assessed demographic and psycho-social factors among young people associated with readiness to be initiated on ART.

**Methods:**

A quantitative cross-sectional study was conducted among newly diagnosed HIV positive young people aged 15–24 years at 4 HIV clinics at Mulago Hospital. Readiness was measured as a self-report by the individual to the question, “How ready do you feel to start ART?

**Results:**

Of the 231 young people enrolled, the mean age (SD) was 20.7years (+/-2.8) and most were female (66.2%). Majority were very ready (53.3%) and very motivated (51.1%) to start ART. Higher treatment readiness was associated with being female (95% CI [5.62, 8.31], p=0.003), thinking that ART cures HIV (95% CI [0.43, 0.86], p=0.005), history of having unprotected sex (95% CI [0.79, 0.87], p=<0.001), anticipating negative HIV results (95% CI [0.26, 0.88], p=0.017), internalized stigma (95% CI [0.83, 0.98], p=0.018) and knowledge of positive ART effects for others (95% CI [0.84, 0.93], p=<0.001).

**Conclusions:**

Understanding the underlying factors associated with ART readiness among young people can inform strategies to support and increase individuals' readiness to initiate ART and early engagement in care.

## Introduction

The burden of HIV/AIDS has reduced globally since the discovery of Antiretroviral Therapy (ART) 1,2. However, eastern and southern Africa continue to be severely affected by the HIV epidemic with about 290,000 new HIV infections among young people aged 15 to 24 years registered in 2018 3. Of these, Uganda contributed 6.6% with national prevalence of 5.7%^4^. Half of Uganda's population are youth 5 and the HIV prevalence among young people aged 15 to 24 years is 3.7%^6^. These young people are one of the key populations who are at a higher risk of acquirinHIV and most of them are unlikely to access care^7^,^8^. Despite several benefits of ART including reduction of AIDS-related mortality, morbidity and transmission, only 73% of people above 15 years are receiving ART 3. It is not clear what percentage of these are young people. Expanding that all people who test positive for HIV start ART is a global public health priority at the heart of the UNAIDS 90-90-90 strategy aimed at maximizing therapeutic and prevention benefits of ART9. However, lack of readiness to start ART has been a major set-back to achieving the set targets with many barriers to ART initiation among young people living with HIV (YPLHIV) such as socio-demographic, structural and economic factors^10^–^17^.

Several studies have suggested that readiness is a key factor in HIV treatment 18–22 and a predictor of ART initiation^23^,^24^. Readiness is defined as “A conscious awareness, on the part of an individual, based on free will that he/she has considered and determined that a particular behavior change (i.e., taking ART as prescribed) will be beneficial” 25. Understanding the relationship between the phenomenon of readiness and subsequent HIV treatment adherence has implications for clinical decision-making and for development of interventions that enhance adherence and prevent HIV drug resistance 26. Today, there is scanty data on factors influencing ART readiness among young people hence a need for more studies.

The 2018 revised national ART treatment guidelines which are in line with the WHO test and treat strategy recommended that all individuals who test positive should be started on ART regardless of their immunological or clinical stage of the disease 27. The guidelines continue to emphasize the need for patients to be ready to adhere well before starting ART, but there are no established, reliable methods for determining pretreatment readiness 27,28. This study assessed the demographic and psycho-social factors associated with readiness to start ART among young people aged 15 to 24 years at four HIV clinics located in Mulago National Referral Hospital in Kampala, Uganda.

## Methods

### Study Design, Setting and Participants

This was a cross sectional quantitative study conducted to assess the relationship between demographic, psychosocial factors and readiness of young people to initiate ART at Four HIV clinics in Mulago National Referral Hospital located in the heart of Kampala city, central Uganda. The 4 clinics included Infectious Diseases Institute (IDI), Mulago ISS clinic, Baylor Uganda and The Aids Support Organization (TASO). These were found to be eligible and included in the study purposely to get an adequate number of respondents. The study involved young people aged 15 – 24 years who had been newly diagnosed as HIV positive either from those clinics or referred for ART from other peripheral health centers.

### Eligibility Criteria

Inclusion criteria were being a young person aged 15 -24 years, newly diagnosed as HIV positive, no current prescription for antiretroviral medication and having given informed consent/assent to participate in the study. Exclusion criteria were being too sick which was determined by a medical officer at the study site and those who asked to withdraw from the study.

### Sample Size and Sampling Procedure

A sample size of 231 participants was calculated with the Kish-Leslie formula 29 using prevalence of readiness (84%) from a readiness study in South Africa^19^ and a margin of error of 5%. The sample size was distributed among the 4 HIV clinics basing on the number of young people (aged 15 to 24 years) that had been initiated on ART from any of these clinics in the previous month as follows; IDI 10, Baylor Uganda 46; Mulago ISS 91 and TASO 84. Consecutive sampling was then used to recruit study participants at each clinic.

### Data Collection

Between February and March 2020, data was collected once from study participants using an interviewer administered structured questionnaire. Prior to the interviews, eligible individuals were selected and given information about the study by trained research assistants. Informed consent and assent for those below 18 years was obtained from all participants prior to participation. Each patient was met individually for about 15 minutes in a private consulting room at the HIV clinics. The investigator stressed to participants that the study was being conducted by Makerere university medical students and was completely independent of the HIV clinic, and that responses were anonymous and would not be seen by any of the staff involved in their care.

### Study Variables

A study tool from a readiness study in South Africa was adopted 19 and made a few modifications using information gathered from a pretest and recommendations from HIV experts to best suite our study context. In addition, the questionnaire was designed to be concise in order to minimize effort among individuals recently diagnosed as HIV-positive.

Readiness to initiate ART was the dependent variable. The test and treat strategy aims at initiating ART on the day of testing if there are no contraindications, and latest within 2 weeks. However, since it doesn't provide an actual measure of readiness, evidence from behavioral research that suggests that individuals can judge their own readiness was used 30. In our study, readiness was measured as a self-report by the individual to the question, “How ready do you feel to start ART?” In addition, the study based on responses to questions designed to measure essential elements of readiness identified in literature that must be present for readiness to exist which include an awareness that treatment will be beneficial, motivation to start treatment and the intention to start treatment soon 18,19,21,31,32.

### Independent Variables

Demographic factors included gender, age, education, marital status, occupation and monthly income. In addition, the study assessed participants' general feeling of their own health in the past week since feeling healthy has been attributed to delay in seeking HIV services 33,34. A question on alcohol use was included as it has been identified as a barrier to HIV treatment among young people 35,36. We included other factors which several studies have directly linked to treatment initiation and adherence such as knowledge about ART^37^, perceived health benefits and knowledge of someone who has experienced positive health effects on ART, denial of being HIV positive 38, concerns about potential adverse effects 39, stigma (both internalized and externalized) 40, social support (from friends and family)41 and disclosure of HIV status^42^. All these psychosocial constructs have been demonstrated to contribute to influencing behavioral change including ART initiation and adherence^20^,^43^–^45^.

### Data management and analysis

The data collected on questionnaires was double entered into Epidata version 4.6.0.2 and then exported to STATA version 15 for cleaning and analysis. Categorical variables were summarized into frequencies and percentages and continuous variables into means and standard deviations since they were normally distributed. Bivariate analysis was done to get the outcome of interest (Readiness to start ART). The variable “how ready do you feel to start ART” was recorded into a binary variable, with the levels, “not at all ready”, “somehow ready” and “don't know” being coded as “not ready” and the variable “very ready” being coded “ready”. Since the outcome was not rare (53.3%), the modified Poisson regression model with clustered standard errors (lustered at facility/clinic levels) was adopted to predict prevalence ratios (PR). All variables that had p-values <0.2, were considered for multivariate analysis.

At multivariate analysis, variables were run in a single model and non-significant variables dropped one at a time starting with the most non-significant, until only significant variables remained in the model. Confounding was tested for by bringing back the variables in the order they were dropped starting with the last dropped. A variable was considered a confounder if the difference between the crude and adjusted prevalence ratios was >10%. All variables were considered to be significant if they had a p-value < 0.05.

### Data Availability

The data underlying the results presented in the study are available from https://doi.org/10.6084/m9.figshare.14812830.v1

## Results

Sample characteristics are presented in (Table 1). Eligible participants (N=231) were screened and enrolled in the study. Majority (39.4%) were tested from the Mulago ISS clinic. The mean age (SD) was 20.7 (+/-2.8) years. Majority of them were female (66.2%), not currently in school (57.6%), had attained secondary school education (47.6%), no history of alcohol use (65.8%) and had unprotected sex before (70%). 50.7% had no income with only 49.8% currently employed. 41.1% of the respondents had children. 46.3% had been in very good health during the last week. Given the high burden of HIV/AIDS and improved access to HIV care facilities in the central region where most of the participants resided, majority reported to have tested for HIV before (91.3%) and knew someone who had died of HIV (60.2%).

**Table 1:** Sample characteristics

Variables	Total N = 231 n	% of Sample
**Enrollment at HIV Clinics**		
IDI	10	4.3
Baylor	46	19.9
Mulago ISS	91	39.4
TASO	84	36.4
Age (years)	Mean = 20.7	SD = 2.8
**Gender**		
Male	78	33.8
Female	153	66.2
**Still in school**	98	42.4
**Education level**		
Primary	51	22.1
Secondary	110	47.6
Tertiary	60	26
None	10	4.3
**Marital status**		
Single	157	68
Married	43	18.6
Separated or divorced	30	13
Widowed	1	0.4
**Employed**	115	49.8
**Monthly Income**		
No salary	117	50.7
<UGX 300,000	83	35.9
>UGX300,000	31	13.4
**Had unprotected sex before**	126	70
**Age (years) when first had sex**	Mean = 17.5	SD = 2.8
**Have children**	95	41.1
**Health in Past week**		
Poor or fair	68	29.4
Good	52	22.5
Very Good	107	46.3
Don't know	4	1.7
**Take alcohol at least once a** **month**	79	34.2
**Repeat HIV testers**	211	91.3
**Has family/Friend who died** **of HIV/AIDS**	139	60.2

N refers to total sample size and n refers to the size of the subset of the sample

UGX 300,000 was equivalent to $82 on 2 February 2020 (at the start of the study)

### Psychosocial characteristics

These are summarized in (Table 2). Majority of the participants had good knowledge about ART i.e. had ever heard about ART (94.4%), knew that ART can't cure HIV (62.3%), ARVs are taken for a lifetime (89.9%) and that ART should be started immediately after diagnosis (83.1%). Perceived HIV risk was common with majority (76.6%) having anticipated to test positive for HIV prior to coming to the clinics. Majority (70.1%) of the respondents knew someone who was on ART and believed that it had a positive health effect on the lives of those people. 32.9% didn't know that they would experience any side effects from ART. Majority reported not to feel at all ashamed (43.3%) or guilty (47.6%) and 41.1% didn't think that they would be treated badly/unfairly by others because of their HIV status. Most participants (35.5%) believed it was not at all likely that they would disclose their HIV status to anyone. Majority reported that they would get social support all the time from their friends (37.7%) and family members (41.1%).

**Table 2:** Psycho-social characteristics of study participants

Variables	Total N = 231 n	% of Sample
**HIV test results anticipation today**		
Positive	177	76.6
Negative	53	22.9
**Knowledge about ART**		
Ever heard of ARVs	218	94.4
ART can't cure HIV	144	62.3
ARVs are taken for a lifetime	205	89.9
ART is started immediately after diagnosis	192	83.1
**How likely to experience side-effects from ARVs?**		
Not at all likely	57	24.7
Somehow likely	53	22.9
Very likely	45	19.5
Don't know	76	32.9
**Knows a friend/family member taking ARVs** **and believes ART had a positive health effect**	162	70.1
**Internalised Stigma**		
Do you feel at all ashamed that you have HIV?		
Not at all	100	43.3
Somehow	61	26.4
Very	20	21.7
Do you feel at all guilty that you have HIV?		
Not at all	110	47.6
Somehow	44	19.1
Very	54	23.4
**Externalized/Perceived Stigma**		
Do you think people will treat you badly/unfairly because you have HIV?		
Not at all	95	41.1
Somehow	55	23.8
Very	46	19.9
**Disclosure**		
How likely do you think it is that you'll tell anyone the results of your HIV test?		
Not at all	82	35.5
Somehow	52	22.5
Very	68	29.4
**Social Support**		
Will get any form of support all the time from;		
Friends	87	37.7
Family members	95	41.1

N refers to total sample size and n refers to the size of the subset of the sample

### Readiness to start ART

More than half of the participants reported to be very ready (53.3%) and very motivated (51.1%) to start ART and only 9.1% were uncertain if they were ready to start ART. In addition, 35.5% were very confident that ART would have a positive health effect on their lives and 35.1% had an intention to start ART within 2 weeks as recommended by the test and treat strategy. The four measures used to assess ART readiness are shown in (Table 3).

**Table 3:** ART readiness among study participants

	Total N = 231 n	% of Sample
**ART readiness components**		
Very ready to start ART	123	53.3
Very motivated to start ART	118	51.1
Very confident that ART will have a positive health effect	82	35.5
Intend to start ART within 2 weeks as per guidelines	81	35.1

N refers to total sample size

n refers to the size of the subset of the sample

### Factors associated with readiness to start ART among young people

Table 4 and Table 5 display results from bivariate and multivariate analysis respectively. The following factors were found to be significantly associated with readiness to start ART among young people at multivariate analysis; being female (aPR=1.22, 95%CI=1.07, 1.39, p=0.003) thinking ART cures HIV (aPR=0.42, 95%CI=0.43, 0.86, p=0.005), having ever had unprotected sex before (aPR=0.83, 95%CI=0.79, 0.87, p=<0.001), anticipating to have negative HIV results (aPR=0.49, 95%CI=0.26, 0.88, p=0.017), knowledge of positive ART effect for others (aPR=0.88, 95%CI=0.84, 0.93, p=<0.001) and feeling somehow ashamed that they have HIV (aPR=0.90, 95%CI=0.83, 0.98, p=0.018). In this model, feeling somehow guilty was found to be a confounder on feeling somehow ashamed.

**Table 4:** Bivariate analysis of factors associated with readiness to start Antiretroviral Therapy

Variables	Not ready n (%)	Ready n (%)	Crude PR	p-value
**Age in years**, mean (SD)	20.3 (2.9)	21.1 (2.7)	1.05	0.306
Female (ref: Male)	68 (63.0)	85 (69.1)	1.14	0.087*
Employed (ref: Not working)	54 (50.0)	61 (49.6)	0.99	0.924
Still in school (ref: No)	55 (50.9)	43 (35.0)	0.73	0.003*
Very good health (ref: poor health)	41 (39.4)	66 (53.7)	1.70	0.013*
Had unprotected sex before (ref: No)	71 (84.5)	55 (57.3)	0.57	0.065*
Have children (ref: No)	43 (39.8)	52 (42.3)	0.95	0.484
Take alcohol at least once a month (ref: No)	14 (13.0)	15 (12.2)	0.95	0.821
Anticipating negative HIV results (ref: positive)	40 (37.4)	13 (10.6)	0.39	<0.001*
Think ART cures HIV (ref: don't)	62 (57.4)	25 (20.3)	0.42	0.002*
ART should be started immediately (ref: later)	78 (72.2)	114 (92.7)	2.57	0.001*
**Internalized stigma**: Likely to feel......(ref: not at all)				
Somehow ashamed	42 (38.9)	19 (15.5)	0.42	<0.001*
Somehow guilty	34 (31.5)	10 (8.1)	0.34	<0.001*
**Externalized stigma**: Likely to be treated badly/unfairly...... (ref: not at all)				
Somehow likely	41 (38.0)	14 (11.4)	0.35	<0.001*
Very Likely	23 (21.3)	23 (18.7)	0.68	0.01*
Knowledge of positive ART effect for others (ref: No)	83 (76.9)	79 (64.2)	1.31	0.088*
**Likelihood of experiencing side effects (ref: not likely)**				
Somehow likely	33 (30.6)	20 (16.3)	0.72	0.362
Very likely	21 (19.4)	24 (19.5)	1.01	0.942
Don't know	27 (25.0)	49 (39.8)	1.23	0.513
**Likely to disclose HIV status to someone (ref: not at all)**				
Somehow likely	28 (25.9)	24 (19.5)	0.77	0.164
Very likely	29 (26.9)	39 (31.7)	0.96	0.787
**Guaranteed social support from... (ref: never)**				
Friends	26 (24.1)	23 (18.7)	0.89	0.196
Family members	30 (27.8)	31 (25.2)	0.78	0.382

n refers to the size of the subset of the sample (N = 231)

Crude PR represents unadjusted Prevalence Ratio

* Only variables that had a p-value <0.2, were considered for multivariate analysis

**Table 5:** Multivariate analysis of factors associated with readiness to start Antiretroviral Therapy

Variables	aPR (95% CI)	p-value
Female (ref: Male)	1.22 (1.07, 1.39)	0.003**
Think ART cures HIV (ref: No)	0.61 (0.43, 0.86)	0.005**
Had unprotected sex before (ref: No)	0.83 (0.79, 0.87)	<0.001**
Anticipating negative HIV results (ref: positive)	0.49 (0.26, 0.88)	0.017**
**Internalized stigma**: Likely to feel......(ref: not at all)		
Somehow ashamed	0.90 (0.83, 0.98)	0.018**
Somehow guilty^a^	0.64 (0.39, 1.04)	0.074
Knowledge of positive ART effect for others (ref: no)	0.88 (0.84, 0.93)	<0.001**

a Confounder

aPR stands for adjusted Prevalence Ratio and CI is confidence interval

** significant variables with a p-value ≤ 0.05

## Discussion

Despite the current clinical guidelines that recommend the immediate start of life saving antiretroviral therapy (ART), HIV infections continue to increase among adolescents and young people 46. Understanding ART readiness among this population will help inform interventions that encourage ART initiation 47.

This study assessed factors associated with readiness to start ART among young people and is among the first studies in Uganda to assess HIV treatment readiness among young people. Results showed that more than half of the respondents were not only very ready and very motivated to start ART but also had intentions to start treatment within less than a month. This is a good indicator that HIV care programs and policy makers should gladly embrace if we are to achieve the second-90 UNAIDS target. These findings should however not make us forget that ART readiness is a dynamic state that changes over time 48, it should therefore be continuously assessed even after treatment initiation. Like other studies done among adults, there was a sizable number of young people who were still uncertain about ART readiness 19,49. We therefore recommend prospective studies to assess ART readiness and interventions to encourage more young people to start ART.

Our study reported that females were more ready to start ART compared to their male counterparts. Other studies have shown that women are more likely to link to care and initiate treatment compared to men^50^–^55^. The other reason could be the difference in health seeking behavior of women compared to that of men. We found that females formed the majority of enrolled patients at the HIV clinics. This finding is in agreement with other studies that found more female enrollment to the HIV clinics than men^56^. We therefore call upon more interventions aimed at bridging this gender gap.

These results showed that several psycho-social factors were directly associated with readiness to start ART. Due to the fact that most of the participants were from the central region with the highest prevalence of HIV/AIDS in Uganda^57^, majority of them knew someone who was living with HIV and taking ART. Knowledge of positive ART benefit/effect for others was another factor associated with ART readiness. This finding is consistent with other studies 19,22,58. Majority of the participants who were very motivated were very likely to start ART. Some of the motivators we identified were having children or knowing someone who was HIV positive and had reported benefits from ART. Other studies have also reported a similar finding 19,48,52. Motivation could further be boosted by good social support and access to supportive health care workers 59. Stigma (both internalized/externalized) has been found to be a barrier to HIV treatment initiation 60.

Our study showed that internalized stigma could be a barrier to starting ART. Efforts to curb all forms of stigma in HIV care should be reinforced to even increase ART readiness because the stigma and discrimination prevalent in today's society have a profound psychological impact from the moment of HIV diagnosis. Participants who believed that ART cures HIV reported to be very ready to start ART. A study by Cohen et al., 2009 reported that some of their participants had a similar belief. This finding suggests that there is need to provide the right information about the benefits of ART during counseling of patients and through other health education channels. It should be emphasized that ART prolongs life rather than cure HIV. Participants who reported a history of having unprotected sex and those anticipating negative HIV test results also reported to be ready to start ART. It is known that having unprotected sex puts one at risk of HIV infection hence we understand that these participants had fear of having contracted the disease and thus the high level of ART readiness. Majority of the participants anticipated a positive HIV test result but they were found not to be ready for treatment. This finding ws associate to denial of HIV diagnosis which has been reported in several studies and has proven to be a huge barrier to ART initiation 62,63.

Results of our study should be considered with limitations. Since readiness is a dynamic parameter that may change at any one time, some individuals may be classified as treatment ready yet they are not. Therefore, HIV clinic staff working with young people are encouraged to continue assessing ART readiness in order to address any barriers to treatment initiation. Secondly, responses to assess readiness were based on the question “How ready do you feel to start ART?” that was asked by the researcher. We are therefore not sure whether these individuals would start ART because they are really ready or because they have been told/asked by the health worker. Research assistants were encouraged to properly explain this question to participants. Thirdly, although interviews were confidential and normalization statements incorporated, there was potential for social desirability bias due to sensitivity of certain questions as well as the fact that data was self-reported hence analysis was done solely on data that was provided by respondents. To address this, interviews were conducted in privacy and made the respondents comfortable by assuring them confidentiality of their responses. Lastly, consecutive sampling has its limitations as a non-probability sampling technique. This was used because the study was done during school time and the HIV clinics register fewer clients (young people) when school is in session compared to holiday time.

## Conclusion

Antiretroviral therapy has proven to be beneficial in prolonging the lives of HIV patients. There continues to be an increasing number of young people living with or acquiring HIV. Therefore, there is need to promote ART initiation through dissemination of the right HIV information to this vulnerable population. This study has shown that young people are ready and motivated to start ART which is a good indicator in this fight against HIV. Winning the fight needs total commitment of all patients right away from the start of treatment. Assessing ART readiness is therefore necessary for therapeutic success. This study has reported the key factors that can be leveraged to ensure timely ART initiation among young people. In addition, new and better tools to assist with determination of ART readiness among young people are needed. Finally, we recommend qualitative and prospective studies in future to better understand this concept of ART readiness.

## References

[1] G Williams B, Lima V, Gouws E (2011). Modelling the Impact of Antiretroviral Therapy on the Epidemic of HIV. Curr HIV Res.

[2] Mahy M, Stover J, Stanecki K, Stoneburner R, Tassie JM (2010). Estimating the impact of antiretroviral therapy: regional and global estimates of life-years gained among adults. Sex Transm Infect.

[3] UNAIDS (2018). AIDSinfo | UNAIDS [Internet].

[4] UNAIDS (2018). Uganda | UNAIDS [Internet].

[5] UBOS (2016). 2014 NPHC-Main Report Main Report National Population and Housing Census 2014 [Internet].

[6] MOF (2013). Millennium Development Goals Report for Uganda 2013. Drivers of MDG Progress in Uganda and Implications for the Post-2015 Development Agenda [Internet].

[7] Bekker LG (2019). HIV control in young key populations in Africa. The Lancet Child and Adolescent Health.

[8] Bowring AL, Ketende S, Rao A, Mfochive Njindam I, Decker MR, Lyons C (2019). Characterising unmet HIV prevention and treatment needs among young female sex workers and young men who have sex with men in Cameroon: a cross-sectional analysis. Lancet Child Adolesc Heal.

[9] UNAIDS (2014). 90-90-90 - An ambitious treatment target to help end the AIDS epidemic [Internet].

[10] Maccarthy S, Saya U, Samba C, Birungi J, Okoboi S, Linnemayr S (2018). “How am I going to live?”: exploring barriers to ART adherence among adolescents and young adults living with HIV in Uganda.

[11] Ammon N, Mason S, Corkery JM (2018). Factors impacting antiretroviral therapy adherence among human immunodeficiency virus-positive adolescents in Sub-Saharan Africa: a systematic review. Public Health.

[12] Croome N, Ahluwalia M, Hughes LD, Abas M (2017). Patient-reported barriers and facilitators to antiretroviral adherence in sub-Saharan Africa. AIDS.

[13] Vreeman RC, Ayaya SO, Musick BS, Yiannoutsos CT, Cohen CR, Nash D (2018). Adherence to antiretroviral therapy in a clinical cohort of HIV-infected children in East Africa. PLoS One [Internet].

[14] Haberer J, Mellins C (2009). Pediatric adherence to HIV antiretroviral therapy [Internet]. Current HIV/AIDS Reports.

[15] Wagner GJ, Linnemayr S, Ghosh-Dastidar B, Currier JS, Hoffman R, Schneider S (2016). Supporting Treatment Adherence Readiness through Training (START) for patients with HIV on antiretroviral therapy: study protocol for a randomized controlled trial. Trials [Internet].

[16] McCauley J, Kern DE, Kolodner K, Dill L, Schroeder AF, DeChant HK (1995). The “battering syndrome”: Prevalence and clinical characteristics of domestic violence in primary care internal medicine practices. Ann Intern Med.

[17] Bassett I V, Regan S, Chetty S, Giddy J, Uhler LM, Holst H (2010). Who starts antiretroviral therapy in Durban, South Africa?. not everyone who should. AIDS [Internet].

[18] Fowler ME (1998). Recognizing the phenomenon of readiness: Concept analysis and case study. J Assoc Nurses AIDS Care.

[19] Maughan-Brown B, Smith P, Kuo C, Harrison A, Lurie MN, Bekker LG (2018). Readiness for Antiretroviral Therapy: Implications for Linking HIV-Infected Individuals to Care and Treatment. AIDS Behav.

[20] Bojan K, Westfall AO, Fernandez MI, Martinez J, Oyedele T, Wilson CM (2019). A measure to assess HIV treatment readiness among adolescents and young adults. Vulnerable Child Youth Stud.

[21] Nordqvist O, Södergård B, Tully MP, Sönnerborg A, Lindblad ÅK (2006). Assessing and achieving readiness to initiate HIV medication. Patient Education and Counseling.

[22] Sylvain H, Delmas P (2012). Readiness in HIV Treatment Adherence: A Matter of Confidence. An Exploratory Study. Open AIDS J.

[23] Balfour L, Tasca GA, Kowal J, Corace K, Cooper CL, Angel JB (2007). Development and validation of the HIV medication readiness Scale. Assessment.

[24] Amuron B, Namara G, Birungi J, Nabiryo C, Levin J, Grosskurth H (2009). Mortality and loss-to-followup during the pre-treatment period in an antiretroviral therapy programme under normal health service conditions in Uganda. BMC Public Health.

[25] Enriquez (2002). An examination of the Index of Readiness as a predictor of adherence and an adherence intervention in HIV+ males who repeatedly failed anti-HIV treatment regimens (Immune deficiency).

[26] Enriquez M, Lackey NR, O'Connor MC, McKinsey DS (2004). Successful adherence after multiple HIV treatment failures. J Adv Nurs.

[27] MOH (2018). CONSOLIDATED GUIDELINES FOR THE PREVENTION AND TREATMENT OF HIV AND AIDS IN UGANDA.

[28] Thompson MA, Aberg JA, Hoy JF, Telenti A, Benson C, Cahn P (2012). Antiretroviral treatment of adult HIV infection: 2012 Recommendations of the International Antiviral Society-USA panel [Internet]. JAMA - Journal of the American Medical Association. JAMA.

[29] Wiegand H, Kish L (1965). Survey Sampling.

[30] Grimes RM, Grimes DE (2009). Patient Readiness to Adhere to HAART. J Int Assoc Physicians AIDS Care [Internet].

[31] Grimes RM, Grimes DE (2010). Readiness: The state of the science (or the Lack Thereof). Current HIV/AIDS Reports.

[32] Sodergard B, Division of Global Health, IHCAR, Karolinska Institutet S (2013). Readiness to start HIV treatment. IJAR [Internet].

[33] Katz IT, Essien T, Marinda ET, Gray GE, Bangsberg DR, Martinson NA (2011). Antiretroviral therapy refusal among newly diagnosed HIV-infected adults. AIDS.

[34] Hachfeld A, Ledergerber B, Darling K, Weber R, Calmy A, Battegay M (2015). Reasons for late presentation to HIV care in Switzerland. J Int AIDS Soc [Internet].

[35] Magidson JF, Fatch R, Orrell C, Amanyire G, Haberer JE, Hahn JA (2019). Biomarker-Measured Unhealthy Alcohol Use in Relation to CD4 Count Among Individuals Starting ART in Sub-Saharan Africa. AIDS Behav [Internet].

[36] Birungi C, Ssembajjwe W, Salisbury TT, Levin J, Nakasujja N, Mpango RS (2020). Substance use among HIV-infected adolescents in Uganda: rates and association with potential risks and outcome factors. AIDS Care - Psychol Socio-Medical Asp AIDS/HIV [Internet].

[37] Hatcher AM, Turan JM, Leslie HH, Kanya LW, Kwena Z, Johnson MO (2012). Predictors of linkage to care following community-based HIV counseling and testing in rural Kenya. AIDS Behav [Internet].

[38] Naik R, Doherty T, Jackson D, Tabana H, Swanevelder S, Thea DM (2015). Linkage to care following a home-based HIV counselling and testing intervention in rural South Africa. J Int AIDS Soc [Internet].

[39] Glendinning E, Spiers J, Smith JA, Anderson J, Campbell LJ, Cooper V (2019). A Qualitative Study to Identify Perceptual Barriers to Antiretroviral Therapy (ART) Uptake and Adherence in HIV Positive People from UK Black African and Caribbean Communities. AIDS Behav [Internet].

[40] Rankin WW, Brennan S, Schell E, Laviwa J, Rankin SH (2005). The Stigma of Being HIV-Positive in Africa. PLoS Med [Internet].

[41] Khamarko K, Janet Myers MJ (2013). The Influence of Social Support on the Lives of HIV-Infected Individuals in Low-and Middle-Income Countries.

[42] Gyamfi E, Okyere P, Enoch A, Appiah-Brempong E (2017). Prevalence of, and barriers to the disclosure of HIV status to infected children and adolescents in a district of Ghana. BMC Int Health Hum Rights [Internet].

[43] Bandura A (1978). Self-efficacy: Toward a unifying theory of behavioral change. Adv Behav Res Ther.

[44] Reynolds NR, Testa MA, Marc LG, Chesney MA, Neidig JL, Smith SR (2004). Factors influencing medication adherence beliefs and self-efficacy in persons naive to antiretroviral therapy: A multicenter, cross-sectional study. AIDS Behav [Internet].

[45] Lee WK, Milloy MJS, Walsh J, Nguyen P, Wood E, Kerr T (2016). Psychosocial factors in adherence to antiretroviral therapy among HIV-positive people who use drugs. Heal Psychol [Internet].

[46] MOH Uganda (2016). Consolidated Guidelines for Prevention and Treatment of HIV in Uganda|Ministry of Health Knowledge Management Portal [Internet].

[47] Johnson MO, Dilworth SE, Stephens E, Lum PJ, Neilands TB (2011). Expectancy and readiness-based predictors of treatment uptake among the Urban Poor Living with HIV. J Acquir Immune Defic Syndr.

[48] Sodergard B (2013). (PDF) Readiness to start HIV treatment [Internet]. IJAR.

[49] Morgenstern TT, Grimes DE, Grimes RM (2002). Assessment of readiness to initiate antiretroviral therapy. HIV Clin Trials.

[50] Auld AF, Shiraishi RW, Mbofana F, Couto A, Fetogang EB, El-Halabi S (2015). Lower levels of antiretroviral therapy enrollment among men with HIV compared with women - 12 Countries, 2002–2013. Morb Mortal Wkly Rep.

[51] Lamb MR, Fayorsey R, Nuwagaba-Biribonwoha H, Viola V, Mutabazi V, Alwar T (2014). High attrition before and after ART initiation among youth (15-24 years of age) enrolled in HIV care. AIDS.

[52] KE M, S N-K, DA M, JT P, GW H (2010). Predictors of Medication Adherence in High Risk Youth of Color Living With HIV. J Pediatr Psychol.

[53] Wolff B, Mbonye M, Coutinho A, Amuron B, Nkabala R, Jaffar S (2009). High levels of psychosocial readiness for ART in an African population at the onset of treatment. SAHARA-J J Soc Asp HIV/AIDS [Internet].

[54] Cornell M, Schomaker M, Garone DB, Giddy J, Hoffmann CJ, Lessells R, Celentano DD (2012). Gender Differences in Survival among Adult Patients Starting Antiretroviral Therapy in South Africa: A Multicentre Cohort Study. PLoS Med [Internet].

[55] UNAIDS (2020). Women are more likely to be on HIV treatment | UNAIDS [Internet].

[56] Kayiso MI, Nakirijja DS, Nassimbwa F (2019). Effectiveness of HIV Linkage to HIV Positive Clients on Treatment in Hospitals in Uganda: The Case of Mengo and Mukono Hospitals HIV Departments. Heal Sci J [Internet].

[57] UPHIA (2016). UGANDA POPULATION-BASED HIV IMPACT ASSESSMENT UPHIA.

[58] Chen WT, Shiu CS, Simoni J, Fredriksen-Goldsen K, Zhang F, Starks H (2009). Attitudes Toward Antiretroviral Therapy and Complementary and Alternative Medicine in Chinese Patients Infected With HIV. J Assoc Nurses AIDS Care.

[59] Van Loggerenberg F, Gray D, Gengiah S, Kunene P, Gengiah TN, Naidoo K (2015). A Qualitative Study of Patient Motivation to Adhere to Combination Antiretroviral Therapy in South Africa. AIDS Patient Care STDS.

[60] CHESNEY MA, SMITH AW (1999). Critical Delays in HIV Testing and Care. Am Behav Sci [Internet].

[61] Cohen CR, Montandon M, Carrico AW, Shiboski S, Bostrom A, Obure A (2009). Association of attitudes and beliefs towards antiretroviral therapy with HIV-seroprevalence in the general population of Kisumu, Kenya. PLoS One.

[62] Nakigozi G, Atuyambe L, Kamya M, Makumbi FE, Chang LW, Nakyanjo N (2013). A Qualitative Study of Barriers to Enrollment into Free HIV Care: Perspectives of Never-in-Care HIV-Positive Patients and Providers in Rakai, Uganda. Biomed Res Int [Internet].

[63] Wringe A, Roura M, Urassa M, Busza J, Athanas V, Zaba B (2009). Doubts, denial and divine intervention: Understanding delayed attendance and poor retention rates at a HIV treatment programme in rural Tanzania. AIDS Care-Psychol Socio-Medical Asp AIDS/HIV.

